# The Role of Social Media in Health Misinformation and Disinformation During the COVID-19 Pandemic: Bibliometric Analysis

**DOI:** 10.2196/48620

**Published:** 2023-09-20

**Authors:** Funmi Adebesin, Hanlie Smuts, Tendani Mawela, George Maramba, Marie Hattingh

**Affiliations:** 1 Department of Informatics University of Pretoria Pretoria South Africa

**Keywords:** bibliometric analysis, COVID-19, fake news, health disinformation, health misinformation, social media

## Abstract

**Background:**

The use of social media platforms to seek information continues to increase. Social media platforms can be used to disseminate important information to people worldwide instantaneously. However, their viral nature also makes it easy to share misinformation, disinformation, unverified information, and fake news. The unprecedented reliance on social media platforms to seek information during the COVID-19 pandemic was accompanied by increased incidents of misinformation and disinformation. Consequently, there was an increase in the number of scientific publications related to the role of social media in disseminating health misinformation and disinformation at the height of the COVID-19 pandemic. Health misinformation and disinformation, especially in periods of global public health disasters, can lead to the erosion of trust in policy makers at best and fatal consequences at worst.

**Objective:**

This paper reports a bibliometric analysis aimed at investigating the evolution of research publications related to the role of social media as a driver of health misinformation and disinformation since the start of the COVID-19 pandemic. Additionally, this study aimed to identify the top trending keywords, niche topics, authors, and publishers for publishing papers related to the current research, as well as the global collaboration between authors on topics related to the role of social media in health misinformation and disinformation since the start of the COVID-19 pandemic.

**Methods:**

The Scopus database was accessed on June 8, 2023, using a combination of Medical Subject Heading and author-defined terms to create the following search phrases that targeted the title, abstract, and keyword fields: (“Health*” OR “Medical”) AND (“Misinformation” OR “Disinformation” OR “Fake News”) AND (“Social media” OR “Twitter” OR “Facebook” OR “YouTube” OR “WhatsApp” OR “Instagram” OR “TikTok”) AND (“Pandemic*” OR “Corona*” OR “Covid*”). A total of 943 research papers published between 2020 and June 2023 were analyzed using Microsoft Excel (Microsoft Corporation), VOSviewer (Centre for Science and Technology Studies, Leiden University), and the *Biblioshiny* package in Bibliometrix (K-Synth Srl) for RStudio (Posit, PBC).

**Results:**

The highest number of publications was from 2022 (387/943, 41%). Most publications (725/943, 76.9%) were articles. *JMIR* published the most research papers (54/943, 5.7%). Authors from the United States collaborated the most, with 311 coauthored research papers. The keywords “Covid-19,” “social media,” and “misinformation” were the top 3 trending keywords, whereas “learning systems,” “learning models,” and “learning algorithms” were revealed as the niche topics on the role of social media in health misinformation and disinformation during the COVID-19 outbreak.

**Conclusions:**

Collaborations between authors can increase their productivity and citation counts. Niche topics such as “learning systems,” “learning models,” and “learning algorithms” could be exploited by researchers in future studies to analyze the influence of social media on health misinformation and disinformation during periods of global public health emergencies.

## Introduction

### Background

The use of social media platforms, including Facebook, Twitter, YouTube, WhatsApp, and Instagram, has revolutionized how we seek information and connect [1]. As of January 2023, there were 4.76 billion social media users worldwide, accounting for 59.4% of the world’s population [2]. Therefore, it was no surprise that many people worldwide relied heavily on social media to seek information about the coronavirus at the height of the COVID-19 pandemic, when governments instituted national lockdowns and restricted movement to contain the spread of the virus [3]. According to Naeem et al [4], global social media use surged from 20% to 87% at the height of the COVID-19 pandemic.

Governments and policy makers worldwide turned to social media to provide relevant information about COVID-19 to their citizens [5,6]. For example, the Macao government (China) used its official Facebook page to disseminate information about COVID-19 to its citizens. This included the government’s plans to combat the virus, dissemination of public health messages aimed at changing behavior, postings aimed at debunking fake news about the virus, and updates about the virus [6]. Health professionals also used social media platforms to rapidly disseminate care guidelines to health workers in different countries, with the guidelines translated into local languages [7].

The viral nature of social media platforms makes them suited to the rapid transmission of credible information, fake news, and unverified information [8]. Although government officials were using social media platforms to disseminate information about COVID-19, peddlers of misinformation and disinformation were exploiting social media to distribute false and unverified information about the virus [4,9].

Misinformation, disinformation, and fake news are not new [10-12] and can be traced back to the Roman Empire [11]. However, the advent of social media, made popular by Web 2.0 technology, means that content creation is no longer restricted to traditional news media. Users are now empowered to become content creators [13], making the widespread distribution of misinformation, disinformation, and fake news a huge challenge for society at large [12].

According to Wardle and Derakhshan [12], misinformation and disinformation are forms of information disorder that involve the dissemination of nonfactual information. The distinguishing feature between the 2 terms is intention. Misinformation entails the distribution of false information without the intention to cause harm. This definition can be expanded to encompass health misinformation, which involves making health-related claims that are not based on scientific evidence without intending to cause harm [14]. In contrast, the motive for disinformation is to deliberately cause harm by sharing false information. A third form of information disorder identified by Wardle and Derakhshan [12] is malinformation, which is the sharing of factual information outside its original context with the intention to cause harm.

Wardle and Derakhshan [12] identified 7 types of mis- and disinformation that lie on a continuum scale that loosely measures the intention to deceive. *Satire or parody* are not intended to cause harm but could be mistaken by some audiences as facts. *Misleading contents* entail the deceptive use of information to manipulate a situation or an issue. *Imposter contents* are disguised to look like or mimic genuine contents, whereas *fabricated contents* are based on nonfactual information with the intention to cause harm. *Content manipulation*, in contrast, involves changing information, images, or videos with the intention to deceive the recipients. In *false content*, factual information is shared with nonfactual contextual information. Finally, *false connection* is when headlines, visuals, or captions are not aligned with the associated content [12].

In contrast, fake news can be described as fabricated content disguised to look like real news [10,15]. According to Tandoc et al [16], fake news can manifest in 6 different forms. *Satire* entails inducing humor based on factual topical issues or critiquing those in power. *Parody* is similar to satire in that it is also intended to induce humor. However, parody is different in that it uses nonfactual or fictitious information to induce humor. *Fabrication* is another manifestation of fake news in which content based on false information is disguised as real news with the intent to misinform the consumers of such content. Fake news can also take the form of *manipulation* of original photos or videos to create incorrect or misleading narratives. Another manifestation of fake news is when *advertisement and public relations* content produced by public relations professionals is included in real news content for financial gain. Although the advertisements may be based on facts, their mashup with real news content could mislead consumers into believing that the entire content is from a real news agency. Finally, fake news can also manifest in the form of *propaganda*, in which contents that may or may not be based on facts are created by a celebrity or public figure with the intention to influence public perception about a particular topic or issue [16].

Despite the overlap between the different manifestations of fake news, misinformation, and disinformation, authors such as Wardle and Derakhshan [12] have refrained from using the term *fake news* because of the complexity of misinformation and disinformation and the propensity of public figures and politicians to misuse *fake news* to refer to critical news items that they do not agree with.

The spread of misinformation, disinformation, and fake news, especially during pandemics, can create panic among citizens and erode their trust in governments and policy makers [17], with the potential for fatal consequences [18]. For example, Soltaninejad [19] reported that misinformation on social media played a role in many Iranian citizens consuming large volumes of methanol in early 2020 as they believed that this would protect them from being infected by the virus. This led to >2000 people being admitted to hospitals across the country with methanol poisoning and 264 subsequent fatalities. In a similar study, Naeem et al [4] analyzed the sources of 1225 fake news stories between January 2020 and April 2020. The authors found that social media platforms accounted for 50% of distributed fake news and identified three main categories of misinformation about the coronavirus: (1) false claims, in which incorrect information about the mode of transmission of COVID-19, the cure for the virus (eg, that consuming large amounts of methanol can cure COVID-19), and the promotion of false prejudices about people of specific ethnic groups being responsible for COVID-19 were being disseminated; (2) the spread of conspiracy theories driven by the pronouncements of some world leaders and public figures about the origin of the virus (eg, that the virus is spread through 5G towers); and (3) the spread of pseudoscientific remedies and treatments purported to be capable of preventing the virus or curing people infected with COVID-19 (eg, that steam inhalation can cure COVID-19).

Another study by Fieselmann et al [20] found that vaccine hesitancy among German citizens could be attributed to the spread of misinformation on Twitter about the benefits of the COVID-19 vaccination. Similarly, the findings by Crouse and Dupuis [18] suggest that people who believe in misinformation and conspiracy theories about COVID-19 have a higher probability of refusing vaccination.

The unprecedented reliance on social media platforms to seek information about the virus [21,22] was accompanied by the dissemination of information about the virus at an alarming rate from both credible and unreliable sources on social media platforms [23]. Social media platforms made it easier to spread misinformation, disinformation, and fake news worldwide [18]. There was an overabundance of factual and nonfactual information [18]. As a consequence, the World Health Organization issued a warning about an impending infodemic and the risk it posed to the global efforts to combat the pandemic [24]. An infodemic can be defined as an overabundance of factual and incorrect information, especially during a pandemic, which could lead to panic and risky behaviors among people [25]. More specifically, Gisondi et al [26] defined the COVID-19 infodemic as “the overwhelming amount of complex and often contradictory information available about COVID-19, inclusive of substantial fake news about the origins of the virus, treatment options unsupported by rigorous clinical data, and baseless claims regarding adverse effects of lifesaving vaccines.” The authors further state that the false narratives about COVID-19 were sometimes propagated by authoritative institutions or public figures who exploited the trust of the general populace to influence their views about, and behaviors toward the virus, with the potential for negative health outcomes.

Several authors [18,26-28] have identified social media platforms as an important driver of the COVID-19 infodemic. Given the huge number of active social media users worldwide [2], it has become much easier to rapidly disseminate information (factual and nonfactual) worldwide. Although humans were generally responsible for sharing and resharing misinformation and disinformation about COVID-19 [18,29], automated web-based accounts, called social bots, have also been identified as superspreaders of the COVID-19 infodemic [28,29]. For example, social bots were responsible for the rapid propagation of COVID-19 misinformation, disinformation, and fake news, including conspiracy theories about the spread of the virus through 5G towers or weakening of the immune system, thus making people more susceptible to the virus, and that Bill Gates was the creator of the virus to obtain financial benefits from the pandemic [30]. In addition to the COVID-19 infodemic, scientific publications related to the virus also surged significantly during the pandemic, with a reduction in the publication of studies that were not related to COVID-19 [31]. For example, a Google Scholar search using the phrase “social media as a driver of misinformation about Covid-19” filtered between 2020 and 2023 yielded 24,500 search results. Given the apparent increase in the number of publications related to COVID-19, it is important to investigate scientific productivity related to the role of social media in health misinformation and disinformation since the start of the COVID-19 pandemic.

### Objectives

The rapid pace at which studies related to the role of social media platforms in driving health misinformation and disinformation about the coronavirus were published at the height of the pandemic makes a bibliometric analysis a suitable research method for this study. Although authors such as Yeung et al [32] have conducted a bibliometric analysis of medical and health-related misinformation on social media, this study is different in that it is specifically focused on the bibliometric analysis of publications related to the role of social media in health misinformation and disinformation during the COVID-19 pandemic. Consequently, this paper reports the results of a bibliometric analysis aimed at answering the following research questions: (1) How have research publications related to the role of social media as a driver of health misinformation and disinformation evolved since the start of the COVID-19 pandemic? (2) What are the trending keywords and niche topics in publications related to the role of social media as a driver of health misinformation and disinformation since the start of the COVID-19 pandemic? (3) Who are the most influential authors of documents and the most influential publishers of studies related to the role of social media as a driver of health misinformation and disinformation since the start of the COVID-19 pandemic? and (4) Who has made up the collaborative networks of authors of publications related to the role of social media as a driver of health misinformation and disinformation since the start of the COVID-19 pandemic?

Studies that report on COVID-19–related health misinformation and disinformation through other media sources such as web-based news and print media are not the focus of this paper.

The remaining sections of the paper are structured as follows. The research design is presented in the *Methods* section. This is followed by the presentation of the results and a discussion of the bibliometric analysis in the *Results* and *Discussion* sections, respectively. Thereafter, the study’s limitations and conclusions are presented in the *Limitations* and *Conclusions* sections, respectively.

## Methods

### Overview

This study used the bibliometric analysis method to investigate the research outputs related to the role of social media as a driver of health misinformation and disinformation since the start of the COVID-19 pandemic. A bibliometric analysis is an objective method of analyzing and synthesizing extant literature to measure the productivity of science, scientists, or scientific activities in a particular field or about a particular topic [33].

### Source Selection Process

Data for the bibliometric analysis were retrieved from the Scopus database on June 8, 2023. Scopus was the database of choice because of its collection of a wide range of abstracts and sources from different disciplines. To ensure that relevant sources were retrieved, we used a combination of Medical Subject Heading and author-defined terms to create the following search phrases, targeting the title, abstract, and keyword fields: (“Health*” OR “Medical”) AND (“Misinformation” OR “Disinformation” OR “Fake News”) AND (“Social media” OR “Twitter” OR “Facebook” OR “YouTube” OR “WhatsApp” OR “Instagram” OR “TikTok”) AND (“Pandemic*” OR “Corona*” OR “Covid*”).

### Source Screening Process

**Figure 1:**
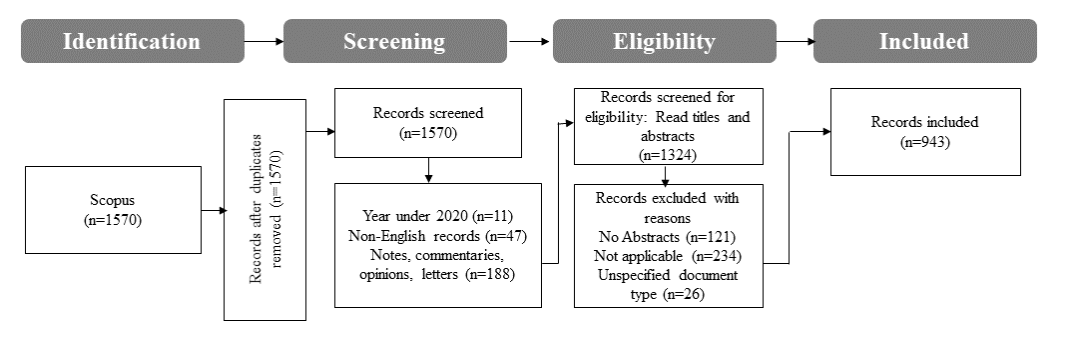
Source screening process.

A total of 1570 search results were retrieved. There were no duplicate documents. The sources were then filtered by limiting them to documents that had been published between 2020 and 2023, resulting in the exclusion of 0.7% (11/1570) of the documents. Thereafter, the sources were filtered by limiting them to documents that were published in English. This resulted in the elimination of 3.01% (47/1559) of the documents. An additional 12.43% (188/1512) of the documents (notes, commentaries, opinions, and letters) were excluded during the screening stage, thereby retaining 1324 documents. The remaining 1324 documents were then screened for eligibility by checking the document types and reading the titles and abstracts. This step resulted in the exclusion of sources with an unspecified document type (26/1324, 1.96%) and 9.14% (121/1324) of the documents, which had no abstract. An additional 17.67% (234/1324) of the documents, which were not relevant to the study (eg, dissemination of fake news about monkeypox, antiviral drugs for smallpox, and social media’s role in panic buying during the pandemic), were excluded. The eligibility screening step resulted in a total of 943 documents being retained as the final set of sources included in the bibliometric analysis. The screening process of the data sources is illustrated in Figure 1, whereas Table 1 summarizes the main information about the sources included in the bibliometric analysis. A copy of the 943 sources included in the bibliometric analysis is provided in Multimedia Appendix 1.

We used Microsoft Excel (Microsoft Corporation) to analyze the number of publications per year, the annual publication growth rate, and document type. VOSviewer (version 1.6.19; Centre for Science and Technology Studies, Leiden University) [34] was used to analyze the keyword co-occurrences and authors’ collaboration networks. Finally, we used the *Biblioshiny* package in Bibliometrix (K-Synth Srl) for RStudio (version 2023.03.0+386 “Cherry Blossom” release; Posit, PBC) [33] to analyze the authors’ number of publications, the most active publishers, the most cited documents, and a thematic map of the topics related to the role of social media in health misinformation and disinformation since the start of the COVID-19 pandemic.

**Table 1:** Main information on sources included in the bibliometric analysis generated using Biblioshiny.

Category and description		Values
Time span		2020-2023
References		44,612
**Authors, n**		
	Authors	4038
	Authors of single-authored documents	88
**Author collaboration**		
	Single-authored documents, n	91
	Coauthors per document, mean	4.77
	International coauthorships (%)	28.31
**Document contents, n**		
	Keywords Plus (ID)	2990
	Author keywords (DE)	1777
**Documents**		
	Total documents, n	943
	Age of document, mean	1.42
	Citations per document, mean	17.4

### Ethical Considerations

We did not apply for ethics clearance for this study because the University of Pretoria only requires ethics clearance for studies that include humans or animals. The bibliometric analysis reported in this study did not include human or animal subjects.

## Results

### Overview

As stated in the *Methods* section, the bibliometric analysis was conducted with the aid of Microsoft Excel, VOSviewer (version 1.6.19) [34], and the *Biblioshiny* package in Bibliometrix for RStudio (version 2023.03.0+386 “Cherry Blossom” release) [33]. *Biblioshiny* is “a shiny app” that provides a web interface for Bibliometrix and can be used by researchers with no programming experience [35]. The results of the bibliometric analysis are presented in the following subsections.

### Annual Publication Growth

A citation analysis of the 943 research papers was conducted to understand how research related to the role of social media in driving health misinformation and disinformation about COVID-19 evolved from 2020 to 2023.

Basic publication growth analysis was conducted in Microsoft Excel using the following annual publication growth rate formula: annual publication growth rate = (number of publications in the year under consideration / number of publications in the preceding year − 1) × 100.

As illustrated in Table 2, a total of 113 documents were published during the first year under consideration (2020). In 2021, there were 306 publications, representing a massive 171% publication growth rate. The number of publications increased to 387 in 2022, representing a 26.4% annual publication growth rate. In 2023, there were only 137 publications as of June 8, 2023, representing a 64.4% decline compared with the preceding year. The decline in the annual publication growth rate can be attributed to the fact that the sources for the bibliometric analysis were extracted on June 8, 2023.

The sources were also analyzed according to their document types. Most of the documents (725/943, 76.9%) were classified as articles, followed by conference papers (107/943, 11.3%). There were 8.3% (78/943) reviews and 3.5% (33/943) book chapters.

The documents were also analyzed from the perspective of the number of publications per author. A total of 4038 authors contributed to the 943 sources that were included in the bibliometric analysis (Table 1). As shown in Table 3, a combined total of 5.3% (50/943) of the documents were published by the top 10 authors. In total, 0.7% (7/943) of the documents were published by the author in the topmost position, whereas 0.5% (5/943) of the documents were published by each of the authors in positions 2 to 8. The authors in positions 9 and 10 published 0.4% (4/943) of the documents each.

Finally, the sources were analyzed according to the publishers to determine the most active ones. The 943 documents were published by 489 unique publishers. Analysis through *Biblioshiny* showed that most of the publishers published only 1 document each. *JMIR*, consisting of *JMIR* (54/943, 5.7%), *JMIR Infodemiology* (22/943, 2.3%), *JMIR Formative Research* (14/943, 1.5%), and *JMIR Public Health Surveillance* (13/943, 1.4%), accounted for 10.9% (103/943) of the publications from the top 10 publishers (Table 4). There were 4.8% (45/943) of publications from the *International Journal of Environmental Research and Public Health*, 2.9% (27/943) from *PLOS ONE*, and 2.4% (23/943) from the *Vaccines* journals. The *Frontiers in Public Health* journal had 1.7% (16/943) of publications, the *Human Vaccines & Immunotherapeutics* journal published 1.5% (14/943), and the *BMC Public Health* journal published 1.4% (13/943).

**Figure 2:**
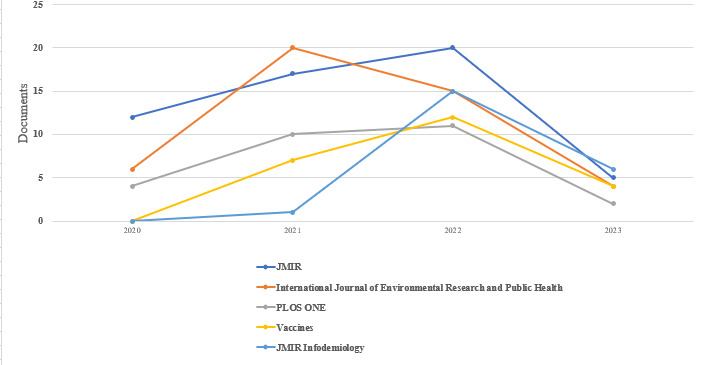
Annual publications per publisher (the year 2023 represents data up until June 2023).

The annual publication, depicted in Figure 2, is only for the top 5 publishers. This is to ensure a meaningful visualization. The line graph corroborates the number of publications by the top 10 publishers shown in Table 4, confirming *JMIR* as the top publisher with 12 documents at the height of the COVID-19 pandemic in 2020. In 2021, the number of publications through *JMIR* increased to 17 (41.7% growth) before peaking at 20 (17.6% growth) in 2022, with only 5 (75% decline) as of June 8, 2023, when the data set was retrieved. The *International Journal of Environmental Research and Public Health* published 6 documents in 2020. This increased to 20 (233% growth) in 2021 before dipping to 15 (25% decline) and 4 (73.3% decline) in 2022 and 2023, respectively. The *PLOS ONE* journal published 4 papers in 2020, a total of 10 (150% growth) in 2021, and 11 (10% growth) in 2022. There were only 2 (81.8% decline) publications from *PLOS ONE* as of June 8, 2023, when the data set was retrieved. There were no publications from the *Vaccines* journal in 2020. This journal published 7 papers in 2021, which increased to 12 (71.4% growth) in 2022. There were only 4 (66.7% decline) publications from the *Vaccines* journal as of June 8, 2023, when the data set was retrieved. In 2020, there were no publications from *JMIR Infodemiology*. The lack of publications by *JMIR Infodemiology* in 2020 can be explained by the fact that the journal was launched in the middle of 2021. Only 1 paper was published in this journal in 2021, also because the journal was launched in the middle of this year. There was a sharp increase to 15 publications (1400% growth) in 2022. There were only 6 (60% decline) publications from *JMIR Infodemiology* as of June 8, 2023, when the data set was retrieved.

**Table 2:** Number of publications per year (the year 2023 represents data up until June 2023).

Year	Publications (n=943), n (%)
2020	113 (11.97)
2021	306 (32.45)
2022	387 (41.04)
2023	137 (14.53)
Total	943 (100)

**Table 3:** Number of publications by the top 10 authors.

Author	Publications (n=943), n (%)
Ahmed	7 (0.7)
Da San Martino	5 (0.5)
Li	5 (0.5)
Liu	5 (0.5)
Luo	5 (0.5)
Nakov	5 (0.5)
Shaban-Nejad	5 (0.5)
Wang	5 (0.5)
Briand	4 (0.4)
Chakraborty	4 (0.4)
Total	50 (5.3)

**Table 4:** Number of publications by the top 10 publishers.

Journal	Publications (n=943), n (%)
JMIR	54 (5.7)
International Journal of Environmental Research and Public Health	45 (4.8)
PLOS ONE	27 (2.9)
Vaccines	23 (2.4)
JMIR Infodemiology	22 (2.3)
Frontiers in Public Health	16 (1.7)
Human Vaccines & Immunotherapeutics	14 (1.5)
JMIR Formative Research	14 (1.5)
BMC Public Health	13 (1.4)
JMIR Public Health and Surveillance	13 (1.4)
Total	241 (25.56)

### Co-Word Analysis

A co-word analysis of the 943 research papers was conducted using VOSviewer to determine the trending keywords in publications related to the role of social media in driving health misinformation and disinformation since the start of the COVID-19 pandemic.

Co-word analysis is a bibliometric analysis technique used to analyze the co-occurrence of keywords in texts and map the strength of the relationships between the keywords [36]. The relationships between the keywords are expressed in terms of the number of occurrences and the total link strength (TLS) of the keywords [34].

**Figure 3:**
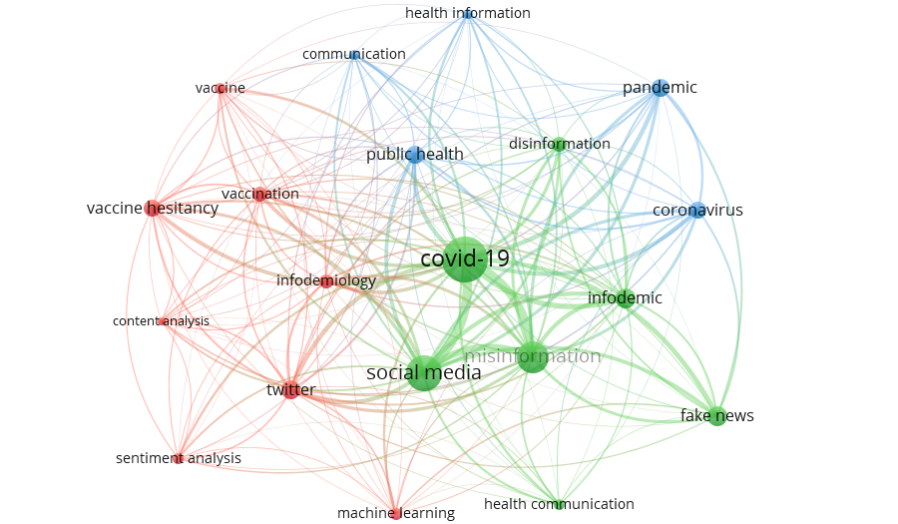
VOSviewer visualization of networks of the top 20 authors’ keywords related to the role of social media in health misinformation and disinformation during the COVID-19 pandemic.

VOSviewer detected 1779 keywords (as opposed to the 1777 detected by *Biblioshiny*) from the 943 documents using the full counting method. Although VOSviewer detected 107 keywords that met the threshold of 5 keywords as the minimum number of occurrences, the number of keywords to be selected was restricted to 20 to ensure the generation of a meaningful visualization of keywords. As illustrated in Table 5, “Covid-19,” “social media,” and “misinformation” were the top 3 keywords, with “Covid-19” having the highest number of occurrences at 566 (TLS=1247), followed by “social media” with 342 occurrences (TLS=956) and “misinformation” with 277 occurrences (TLS=797). It should be noted that the higher the value of the TLS for a keyword, the stronger the link [34] and the higher the ranking assigned to a keyword. Figure 3 illustrates the top 20 authors’ keyword networks. The size of each circle in the network is an indication of the number of keyword occurrences. The connections (links) between keywords indicate the strength of their relationships with one another.

**Table 5:** Top 20 keywords according to total link strength (TLS) using VOSviewer.

Keyword rank	Keyword	Number of occurrences	TLS
1	“Covid-19”	566	1247
2	“Social media”	342	956
3	“Misinformation”	277	797
4	“Infodemic”	114	385
5	“Twitter”	96	362
6	“Public health”	89	283
7	“Pandemic”	86	275
8	“Coronavirus”	84	272
9	“Fake news”	111	268
10	“Infodemiology”	52	262
11	“Vaccine hesitancy”	82	226
12	“Vaccination”	62	210
13	“Disinformation”	65	202
14	“Health information”	31	128
15	“Vaccine”	35	120
16	“Communication”	29	118
17	“Content analysis”	21	107
18	“Sentiment analysis”	37	106
19	“Health communication”	37	105
20	“Machine learning”	38	97

### Coauthorship and Collaboration by Country

To understand the collaboration networks of authors from different countries in publications related to the role of social media in driving health misinformation and disinformation since the start of the COVID-19 pandemic, the country of affiliation of the coauthored documents was analyzed using VOSviewer (version 1.6.19).

Of the 111 countries detected by VOSviewer, 51 (45.9%) met the minimum threshold of 5 documents from a country with at least 1 citation from that country. Table 6 illustrates the top 20 coauthorships among countries. As shown in Table 6, authors from the United States had the highest number of collaborations, with 311 coauthored documents. The collaborations between the United States and other countries yielded the highest number of citations (n=5388), with a TLS of 141. The higher the TLS, the stronger the collaboration between authors [34]. South Africa and Nigeria were the only African countries in the top 20 coauthorship list. The 2 countries were ranked 10th (coauthorship=26; TLS=32) and 17th (coauthorship=26; TLS=26), respectively, in their collaborations with other countries.

**Figure 4:**
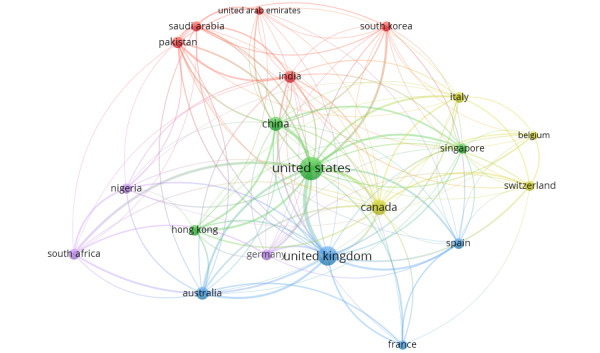
VOSviewer visualization of the top 20 countries’ collaboration networks on publications related to the role of social media in health misinformation and disinformation during the COVID-19 pandemic.

**Figure 5:**
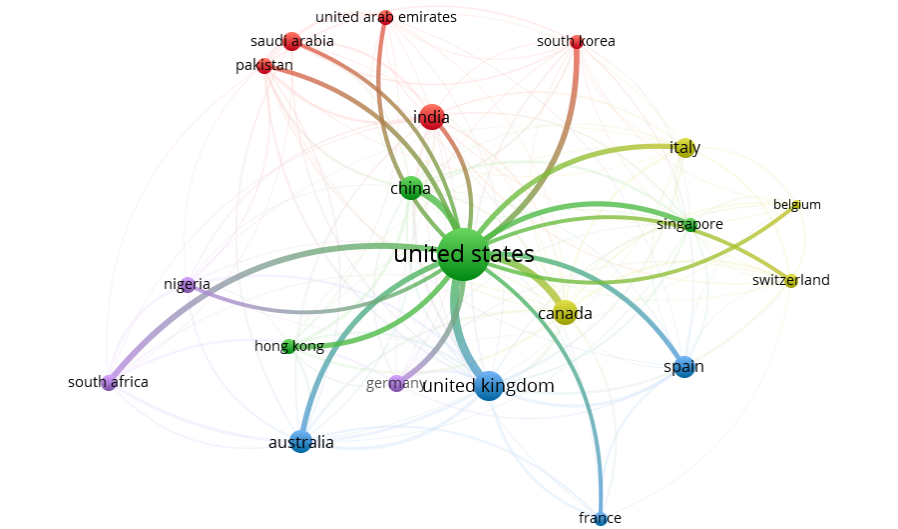
VOSviewer visualization accentuating the collaboration networks between the United States and other countries on publications related to the role of social media in health misinformation and disinformation during the COVID-19 pandemic.

**Figure 6:**
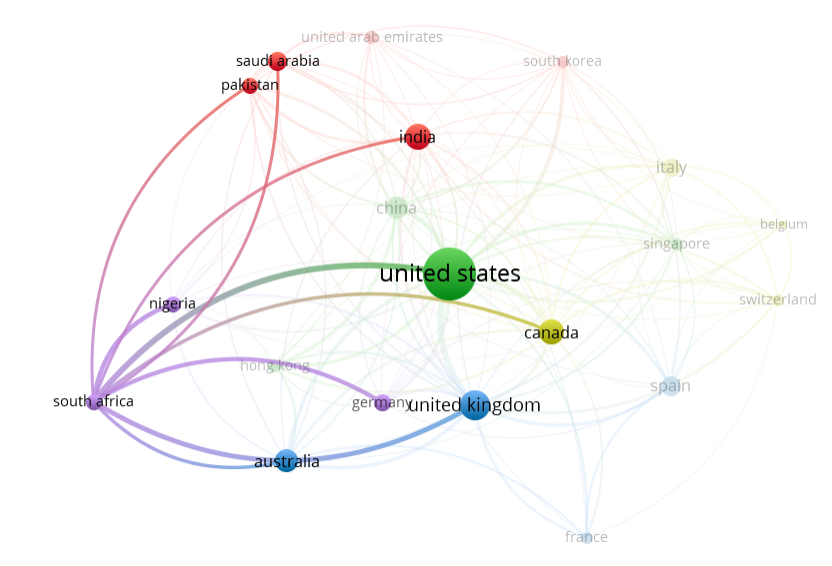
VOSviewer visualization accentuating the collaboration networks between South Africa and other countries on publications related to the role of social media in health misinformation and disinformation during the COVID-19 pandemic.

Figure 4 depicts the collaboration networks among the top 20 countries on topics related to the role of social media on health misinformation and disinformation during the COVID-19 pandemic. The collaboration networks between the United States and other countries, as well as between South Africa and other countries, are accentuated in Figures 5 and 6, respectively.

**Table 6:** Top 20 coauthorships by country using VOSviewer.

Country rank	Country	Number of documents	Total citations, n	TLS^a^
1	United States	311	5388	141
2	United Kingdom	94	2350	99
3	Canada	66	2021	64
4	China	61	987	54
5	Australia	54	1505	44
6	India	70	788	40
7	Pakistan	24	713	34
8	Hong Kong	21	463	32
9	Italy	42	1011	32
10	South Africa	26	86	32
11	Germany	30	278	31
12	Spain	50	1012	30
13	Singapore	18	232	28
14	South Korea	18	128	28
15	Switzerland	17	406	28
16	Saudi Arabia	35	376	27
17	Nigeria	26	137	26
18	France	19	190	23
19	Belgium	7	301	18
20	United Arab Emirates	20	198	17

^a^TLS: total link strength.

### Most Cited Documents

To determine the authors with the most impactful publications, the top 20 most cited publications related to the role of social media in driving health misinformation and disinformation since the start of the COVID-19 pandemic were analyzed.

One of the indicators of a scientific publication’s impact is the number of citations of an author’s published research. The influence of the authors of the 943 research papers included in the bibliometric analysis was measured using the global citation feature in *Biblioshiny* and the impact factor of the journals in which the research papers were published. All the top 20 most cited papers were coauthored by at least 2 authors. The paper with the highest number of citations (n=522) was written by Puri et al [37], followed by the paper by Romer and Jamieson [38] in the second position with 491 citations and the paper by Islam et al [39] with 461 citations in the third position. All the top 20 most cited papers were published in journals with impact factors >3, with 3.7 being the lowest impact factor and 30.8 being the highest (Table 7).

**Table 7:** Top 20 most cited papers.

Study, year	Title	Total citations (Scopus), n	Journal	Journal impact factor^a^
Puri et al [37], 2020	Social Media and Vaccine Hesitancy: New Updates for the Era of COVID-19 and Globalized Infectious Diseases	522	Human Vaccines & Immunotherapeutics	4.52
Romer and Jamieson [38], 2020	Conspiracy Theories as Barriers to Controlling the Spread of COVID-19 in the U.S.	491	Social Science & Medicine	5.4
Islam et al [39], 2020	COVID-19-Related Infodemic and its Impact on Public Health: A Global Social Media Analysis	461	American Journal of Tropical Medicine and Hygiene	3.7
Abd-Alrazaq et al [40], 2020	Top Concerns of Tweeters During the COVID-19 Pandemic: A Surveillance Study	442	JMIR	7.08
Ahmed et al [41], 2020	COVID-19 and the 5G Conspiracy Theory: Social Network Analysis of Twitter Data	385	JMIR	7.08
Tasnim et al [42], 2020	Impact of Rumors and Misinformation on COVID-19 in Social Media	380	Journal of Preventive Medicine & Public Health	3.97
Allington et al [43], 2021	Health-Protective Behaviour, Social Media Usage and Conspiracy Belief During the COVID-19 Public Health Emergency	350	Psychological Medicine	6.03
Ahmad and Murad [44], 2020	The Impact of Social Media on Panic During the COVID-19 Pandemic in Iraqi Kurdistan: Online Questionnaire Study	348	JMIR	7.08
Hua and Shaw [45], 2020	Corona Virus (Covid-19) “Infodemic” and Emerging Issues Through a Data Lens: The Case of China	315	IJERPH^b^	4.61
Li et al [21], 2020	YouTube as a Source of Information on COVID-19: A Pandemic of Misinformation?	315	BMJ Global Health	8.05
Tsao et al [46], 2021	What Social Media Told Us in the Time of COVID-19: a Scoping Review	279	The Lancet Digital Health	30.8
Laato et al [47], 2020	What Drives Unverified Information Sharing and Cyberchondria During the COVID-19 Pandemic?	250	European Journal of Information Systems	9.01
Tangcharoensathien et al [48], 2020	Framework for Managing the COVID-19 Infodemic: Methods and Results of an Online, Crowdsourced WHO Technical Consultation	247	JMIR	7.08
van der Linden et al [49], 2020	Inoculating Against Fake News About COVID-19	219	Frontiers in Psychology	3.9
Gallotti et al [50], 2020	Assessing the Risks of “Infodemics” in Response to COVID-19 Epidemics	205	Nature Human Behaviour	24
Suarez-Lledo and Alvarez-Galvez [14], 2021	Prevalence of Health Misinformation on Social Media: Systematic Review	201	JMIR	7.08
Islam et al [51], 2021	COVID-19 Vaccine Rumors and Conspiracy Theories: The Need for Cognitive Inoculation Against Misinformation to Improve Vaccine Adherence	199	PLOS ONE	3.75
Malecki et al [52], 2021	Crisis Communication and Public Perception of COVID-19 Risk in the Era of Social Media	198	Clinical Infectious Diseases	11.8
Jennings et al [53], 2021	Lack of Trust, Conspiracy Beliefs, and Social Media Use Predict COVID-19 Vaccine Hesitancy	173	Vaccines	7.8
Barua et al [54], 2020	Effects of Misinformation on COVID-19 Individual Responses and Recommendations for Resilience of Disastrous Consequences of Misinformation	166	Progress in Disaster Science	6.3

^a^2022 journal impact factor.

^b^IJERPH: International Journal of Environmental Research and Public Health.

### Thematic Map of Keywords

**Figure 7:**
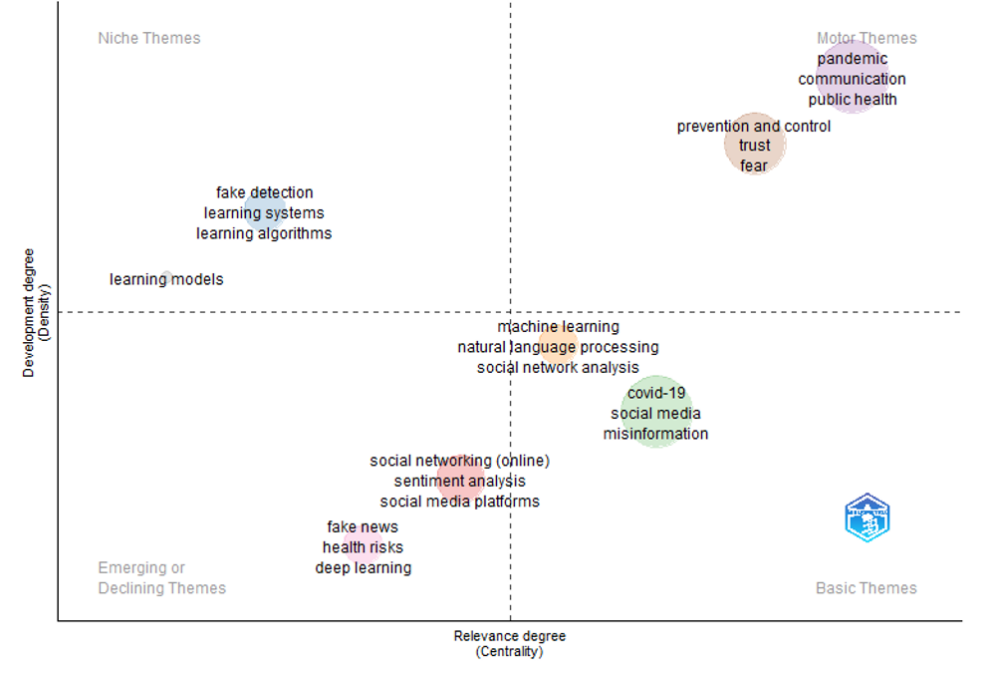
Biblioshiny visualization of thematic mapping of keywords related to the role of social media in health misinformation and disinformation during the COVID-19 pandemic.

Finally, the *Biblioshiny* package in Bibliometrix for RStudio was used to generate the thematic map illustrated in Figure 7. The thematic map provides a 2D view of the degree of development of the themes, which is computed by *Biblioshiny* based on the external links (centrality) and internal strengths (density) of the keywords in the documents that were analyzed [55,56]. The *Biblioshiny* package has been used by many authors, including Wilczewski and Alon [55], Di Cosmo et al [56], and Bretas and Alon [57], to derive a conceptual thematic map for a specific research topic. We used the Keyword Plus feature in *Biblioshiny* based on 250 units of keywords, with a minimum cluster frequency of 5 per 1000 document units and 3 labels per cluster. To ensure a meaningful visualization, basic keywords such as “human,” “female,” “male,” “article,” and “aged” were removed from the set of keywords that were used by *Biblioshiny* to generate the conceptual thematic map. The following words were also merged with their synonyms: (1) “Coronavirus disease 2019,” “coronavirus infection,” “coronavirus infections,” “coronaviruses,” and “sars-cov-2” were merged with “Covid-19”; (2) “Covid-19 vaccines,” “vaccination,” “sars-cov-2 vaccine,” “vaccine,” and “vaccines” were merged with “covid-19 vaccine”; and (3) “Pandemics” was merged with “pandemic.”

As illustrated in Figure 7, there are 4 quadrants on the thematic map [55,56] (Textbox 1).

**The 4 quadrants on the thematic map.**

- Topics in the motor themes (the upper right quadrant) have high density and centrality. Topics in this quadrant are seen as the mainstream topics in a specific field of research.
- Topics in the niche themes (the upper left quadrant) have high density but low centrality. This implies that topics in this quadrant are seen as specialized topics in a field of research.
- Topics in the basic themes (the lower right quadrant) have low density but high centrality. This implies that topics in this quadrant are general themes in a field of research.
- Topics in the emerging or declining themes (the lower left quadrant) have low density and centrality. This implies that topics in this quadrant are weakly developed themes in a field of research.

As shown in Figure 7, there are 2 clusters of *motor themes*. The first is “pandemic,” “communication,” and “public health,” whereas the second is “prevention and control,” “trust,” and “fear.”

There are 2 clusters of *basic themes.* The first cluster is “machine learning,” “natural language processing,” and “social network analysis.” This could imply that an increasing number of publications included in the bibliometric analysis used these techniques to detect health misinformation and disinformation on social media platforms during the COVID-19 pandemic. As expected, “Covid-19,” “social media,” and “misinformation” were the dominant topics in the second cluster of the *basic themes* quadrant. This is not a surprise given that these topics are the focus of this bibliometric analysis.

The *niche themes* quadrant also consists of 2 clusters. The first is “fake detection,” “learning systems,” and “learning algorithms,” whereas the second cluster has only 1 item, “learning models.” As stated earlier, topics that appear in the *niche themes* quadrant are generally seen as specialized topics in a research area [55,56]. This could imply that these topics and techniques were not widely used by the authors of the documents included in the bibliometric analysis.

Finally, the *emerging or declining themes* quadrant also has 2 clusters of themes. The first is “social networking (online),” “sentiment analysis,” and “social media platforms.” The second is “fake news,” “health risks,” and “deep learning.”

## Discussion

### Principal Findings

The bibliometric analysis of studies related to the role of social media in health misinformation and disinformation since the start of the COVID-19 pandemic revealed a high level of publications by authors from countries worldwide, with 4038 authors involved in the 943 documents analyzed. However, most of the publications were by authors from high-income countries. The study also showed a high level of global collaboration, with the highest level between authors from the United States and those from other countries. The study showed that the higher the number of documents published through collaborations, the higher the total number of citations of the documents. This implies that a high level of collaboration can increase the productivity of researchers and the citation counts of their publications. As reported by Abramo et al [58] and Ceballos et al [59], collaboration between researchers has a positive influence on their productivity.

Keywords such as “Covid-19,” “social media,” “misinformation,” and “infodemic” featured prominently in the 943 documents that were analyzed. The prominence of these keywords was no surprise given the focus of the bibliometric analysis. The study revealed important keyword themes that are related to the role of social media in health misinformation and disinformation since the outbreak of COVID-19. Basic topics that included “Covid-19,” “social media,” and “misinformation” were revealed. The analysis also showed that techniques such as “machine learning,” “social network analysis,” and “natural language processing” were used by researchers to detect health misinformation and disinformation on social media platforms during the COVID-19 pandemic. In addition, niche topics such as “learning systems,” “learning models,” and “learning algorithms” were revealed through the *Biblioshiny* thematic mapping of keywords. According to Wilczewski and Alon [55] and Di Cosmo et al [56], terms or topics that appear in the *niche themes* quadrant of *Biblioshiny*’s thematic map are seen as specialized topics in a research area.

The bibliometric analysis showed that the publication by Puri et al [37], “Social media and vaccine hesitancy: new updates for the era of COVID-19 and globalized infectious diseases,” was the document with the highest number of citations (n=522). This was followed by the publication by Romer and Jamieson [38] titled “Conspiracy theories as barriers to controlling the spread of COVID-19 in the US,” cited 491 times. The paper by Islam et al [39], titled “COVID-19-related infodemic and its impact on public health: A global social media analysis,” was the third most cited with 461 citations. These top 3 most cited papers were published by authors from high-income countries (Canada, the United States, and the United Kingdom), Asia, and the Middle East. In addition, *JMIR* as an umbrella journal had the highest number of publications.

The research reported in this paper is similar to that of Yeung et al [32] in that both studies used the bibliometric analysis method. These 2 studies are also related to social media and health misinformation and disinformation. However, this study is different in that it is specifically concerned with the analysis of research papers that focused on social media and health misinformation and disinformation during the COVID-19 pandemic. In addition, the *Biblioshiny* tool that we used is a methodological contribution to additional tools that can be used by researchers when conducting bibliometric analysis.

Although the worst of the COVID-19 pandemic appears to be behind us, similar outbreaks could reoccur. For example, an outbreak of monkeypox was reported in May 2022 [60]. The findings of this bibliometric analysis can be leveraged by other researchers through studies that investigate how niche topic areas (“learning systems,” “learning algorithms,” and “learning models”) can be used to analyze the influence of social media on health misinformation and disinformation during periods of global public health emergencies.

### Limitations

The results of the bibliometric analysis reported in this paper are based on the documents retrieved from the Scopus database on June 8, 2023. As such, the validity of the results is only based on the sources that were analyzed. Other relevant studies that were not available through the Scopus database could have been excluded. Hence, the results are not necessarily a full representation of studies related to the role of social media in health misinformation and disinformation since the outbreak of the COVID-19 pandemic.

### Conclusions

This bibliometric analysis aimed to understand scientific productivity on topics related to the role of social media in health misinformation and disinformation since the start of the COVID-19 pandemic. A total of 943 publications between 2020 and June 8, 2023, were included in the bibliometric analysis. This study revealed a number of key findings. Our analysis showed that the highest number of publications related to the role of social media in health misinformation and disinformation since the start of the COVID-19 pandemic was 387 in 2022. We also found that *JMIR* was the most productive publisher with a combined 10.9% (103/943) of the publications. The results of our analysis also showed that “Covid-19,” “social media,” and “misinformation” were the top 3 keywords, with 566, 342, and 277 occurrences, respectively. This was corroborated by the thematic mapping of keywords through *Biblioshiny*, which identified “Covid-19,” “social media,” and “misinformation” as one of the clusters of topics of the basic themes related to the role of social media in health misinformation and disinformation during the COVID-19 outbreak. In addition, the terms “learning systems,” “learning models,” and “learning algorithms” were revealed as niche topics on the role of social media in health misinformation and disinformation during the COVID-19 outbreak.

Finally, the study revealed that authors from the United States had the highest number of collaborations with authors from different parts of the world in publishing research papers related to the role of social media in health misinformation and disinformation during the COVID-19 outbreak, whereas the research paper by Puri et al [37] titled “Social media and vaccine hesitancy: new updates for the era of COVID-19 and globalized infectious diseases” had the highest number of citations at 522.

### Future Research

Niche topics offer opportunities for researchers in new areas that can be exploited to analyze the influence of social media on health misinformation and disinformation during periods of global public health emergencies.

## Appendix

Multimedia Appendix 1 Data source.

## Abbreviations

TLS: total link strength
